# Data for outcomes of acute hospital administration of amiodarone and/or lidocaine in shockable patients presenting with out-of-hospital cardiac arrest

**DOI:** 10.1016/j.dib.2016.11.085

**Published:** 2016-11-30

**Authors:** Chien-Hua Huang, Ping-Hsun Yu, Min-Shan Tsai, Po-Ya Chuang, Tzung-Dau Wang, Chih-Yen Chiang, Wei-Tien Chang, Matthew Huei-Ming Ma, Chao-Hsiun Tang, Wen-Jone Chen

**Affiliations:** Department of Emergency Medicine and Department of Internal Medicine (Cardiology), College of Medicine, National Taiwan University, Taipei, Taiwan

## Abstract

The data presented in this article are related to the research article entitled “Acute Hospital Administration of Amiodarone and/or Lidocaine in Shockable Patients Presenting with Out-of-hospital Cardiac Arrest: A Nationwide Cohort Study” (C.H. Huang, P.H. Yu, M.S. Tsai et al., 2016) [1]. The data contains the information of co-morbidities coding from ICD-9 CM codes and specific difference in requirement between medical centers and non-medical centers in resuscitation. Univariate and multivariate logistic regression analysis for factors related to the outcome of survival to ICU admission and survival to hospital discharge are included in the data set. The data also contains bootstrap sensitivity analysis of the logistic regression model for survival to ICU admission and hospital discharge outcomes in out-of-hospital cardiac arrest. Subgroup analysis of epinephrine dosage related to outcome of one-year survival is shown.

**Specifications Table**

**Table:** 

Subject area	*Biology*
More specific subject area	*Acute cardiac care*
Type of data	*Tables*
How data was acquired	*Data analysis for national health insurance database*
Data format	*Analyzed*
Experimental factors	*Data are analyzed to figure out the outcomes related variables*
Experimental features	*Retrospective, observational, and nationwide population-based cohort study of patients with non-traumatic cardiac arrest*
Data source location	*A nationwide cohort study in Taiwan*
Data accessibility	*The analyzed data is with this article.*

**Value of the data**

• The data provide information the ways of coding co-morbidities and hospital levels in the resuscitation study. The short term outcomes of survival to hospital admission, intermediate outcome of survival to hospital discharge are important in cardiac arrest patient.
• The data provides the information so that the effects of specific intervention can be comprehensively figured out and compared.
• Subgroup analysis of patients with different dosage of epinephrine used in resuscitation show the interaction with effects of anti-arrhythmic agents.

## 1 Data

The data contains the information of co-morbidities coding from ICD-9 CM codes and specific difference in requirement between medical centers and non-medical centers in resuscitation as shown in Table 1, Table 2. Univariate and multivariate logistic regression analysis for factors related to the outcome of survival to ICU admission and survival to hospital discharge are included in the data set Table 3a, Table 3b. The data also contains bootstrap sensitivity analysis of the logistic regression model for survival to ICU admission and hospital discharge outcomes in out-of-hospital cardiac arrest as shown in Table 4. Subgroup analysis of epinephrine dosage related to outcome of one-year survival is shown in Table 5.

## 2 Experimental design, materials and methods

Medical records/reports accruing between years 2004 and 2011 were retrieved from the Taiwan National Health Insurance Research Database (NHIRD) for review. This repository releases anonymous secondary data for research purposes and houses all claims data from the National Health Insurance (NHI) program in Taiwan. Launched in 1995, the NHI provides coverage for >99% of the entire Taiwanese population of 23.74 million [2]. The database details all patient demographics and orders for medical care. Taiwan׳s NHI Bureau is responsible for comprehensive review of medical records and examination reports [3]. Disease diagnoses are coded according to the International Classification of Disease, Ninth Revision, Clinical Modification (ICD-9-CM). The study protocol was approved by the National Taiwan University Hospital Research Ethics Committee.

## 3 Study design

This retrospective, observational, and nationwide population-based cohort study of patients with non-traumatic cardiac arrest was designed to investigate the impact of amiodarone and lidocaine usage on survival outcomes. Subjects were selected entirely from the NHIRD, all undergoing DC shock and cardiopulmonary resuscitation during short emergency room stay between January, 2004 and December, 2011. Grounds for exclusion were stipulated as follows: 1) age <18 years, 2) trauma-related event, 3) emergency room stay >6 h, or 4) non-level one triage. Patients were categorized and triaged into level-one if vital signs were extremely unstable and needed immediate resuscitation when presented to emergency department. Any known recipients of lidocaine or amiodarone (oral or intravenous) within 1 year previously were also excluded to minimize therapeutic interference. Patients were followed from cardiac arrest index date to 1-year survival status or death. Analysis was based on data from emergency rooms and hospitalization and not from ambulance or from resuscitation on the scene in the study [1].

## Figures and Tables

**Table 1:** Co-morbidities coding from ICD-9 CM codes.

Co-morbidities	ICD-9 CM codes
Diabetes mellitus	250.*
Hypertension	401.*, 402.*, 403.*, 404.*, 405.*
Coronary artery disease	410.*, 411.*, 412.*, 413.*, 414.*
Congestive heart failure	428.*
Atrial fibrillation	427.31
Chronic kidney disease	585
Malignancy	140.*~172.*, 174.*~194*, 200.*~208.*
Chronic obstructive pulmonary disease	491.*, 492.*, 494.*, 496.*
Asthma	493.*

**Table 2:** Specific difference in requirement between medical centers and non-medical centers in resuscitation.

	Medical centre	Non-medical centre
Chief of emergency department	Emergency medicine specialist	Any medical specialist
Physician qualification	1. 70% of total physicians are fixed in ED 2. More than 50% of total fixed physician are emergency medicine specialist	1. 30% of total physicians are fixed in ED 2. Any medical specialist
Qualified advanced cardiac life support (ACLS) training	More than 75% of total stuff (including physicians and nurses)	More than 50% of total stuff (including physicians and nurses)
Management for acute coronary syndrome	Cardiologist and cardiovascular surgeon consultation at any time	Cardiologist consultation at any time
Perform percutaneous coronary intervention	1. Always available at any time	1. Not always available
	2. Door-to-balloon time <90 min in 75% of total STEMI patients	2. Transfer the patient if PCI is not available

**Table 3a:** Univariate and multivariate logistic regression analysis for factors related to the outcome of survival to ICU admission.

	Univariate		Multivariate	
	OR (95% CI)	*P* value	OR (95% CI)	*P* value
Age (pear year)	0.99(0.99~0.99)	<0.0001	0.99 (0.98~0.99)	<0.0001
Male	0.92(0.87~0.98)	0.0141	0.91(0.85~0.98)	0.01
Medication use				
Both	2.82(2.52~3.17)	<0.0001	4.05(3.56~4.61)	<0.0001
Amiodarone	2.04(1.90~2.18)	<0.0001	2.23(2.07~2.41)	<0.0001
Lidocaine	1.87(1.62~2.16)	<0.0001	2.32(1.99~2.71)	<0.0001
Neither	1		1	
Urbanization level				
1	1.35(1.24~1.47)		1.23(1.12~1.36)	<0.0001
2	1.41(1.30~1.52)		1.26(1.16~1.37)	<0.0001
3	1.22(1.10~1.37)		1.36(1.21~1.53)	<0.0001
4	1		1	
CCI	0.98(0.97~0.99)	<0.0001		
Pre-existing medical disease^a^				
DM	1.06(0.9956~1.14)	0.06		
Hypertension	0.93(0.87~0.98)	0.01		
CAD	0.88(0.82~0.95)	0.0007	0.89(0.82~0.97)	0.005
HF	0.94(0.86~1.03)	0.18		
Af	1.16(0.99~1.35)	0.06	1.20(1.01~1.41)	0.03
CKD	1.16(1.05~1.28)	0.0024		
Malignancy	0.93(0.83~1.03)	0.17	0.88(0.78~0.98)	0.02
COPD	0.80(0.73~0.88)	<0.0001		
Asthma	0.85(0.75~0.97)	0.01		
Year of events				
2004	1		1	
2005	1.17(1.03~1.33)	0.01	1.19(1.04~1.35)	0.01
2006	1.54(1.37~1.74)	<0.0001	1.50(1.32~1.70)	<0.0001
2007	1.54(1.36~1.74)	<0.0001	1.48(1.30~1.69)	<0.0001
2008	1.82(1.61~2.05)	<0.0001	1.75(1.54~1.99)	<0.0001
2009	1.78(1.57~2.01)	<0.0001	1.81(1.59~2.06)	<0.0001
2010	1.82(1.61~2.07)	<0.0001	1.78(1.56~2.03)	<0.0001
2011	1.73(1.52~1.96)	<0.0001	1.72(1.50~1.97)	<0.0001
Epinephrine dose (per mg)	0.90(0.89~0.90)	<0.0001	0.87(0.87~0.88)	<0.0001
Vasopressin use	2.14(0.97~4.48)	0.03	2.86(1.35~6.06)	0.006
Resuscitation in medical centre	1.70(1.58~1.82)	<0.0001	1.60(1.47~1.74)	<0.0001

Regression to age, gender, underlying diseases, Charlson comorbidity index, epinephrine dose, vasopressin use, hospital level, urbanization level, year of event.

OD: odds ratio, CI: confidence interval.

a CCI: Charlson comorbidity index; DM: diabetes mellitus; CAD: coronary artery disease; HF: heart failure; Af: atrial fibrillation; CKD: chronic kidney disease; COPD: chronic obstructive pulmonary disease.

**Table 3b:** Univariate and multivariate analysis for factors related to the outcome of survival to hospital discharge.

	Univariate			Multivariate	
	OR (95% CI)		*P* value	OR (95% CI)	*P* value
Age, pear year	0.98(0.98~0.98)		<0.0001	0.99(0.98~0.99)	<0.0001
Male	1.16(1.03~1.30)		0.01		
Medication					
Both	4.46(3.76~5.28)		<0.0001	4.26(3.36~5.41)	<0.0001
Amiodarone	3.08(2.74~3.45)		<0.0001	2.79(2.40~3.24)	<0.0001
Lidocaine	2.66(2.11~3.35)		<0.0001	2.51(1.88~3.36)	<0.0001
Neither	1			1	
Urbanization Level					
1	1.50 (1.29~1.73)		<0.0001		
2	1.35 (1.18~1.55)		<0.0001		
3	1.13 (0.93~1.38)		0.23		
4	1				
CCI	0.88(0.86~0.90)		<0.0001	0.95(0.92~0.99)	0.005
Pre-existing medical disease^a^					
DM		0.77(0.68~0.88)	<0.0001		
Hypertension		0.80(0.71~0.89)	<0.0001		
CAD		0.81(0.71~0.93)	0.0026		
heart failure		0.86(0.73~1.00)	0.052		
Af		0.90(0.66~1.21)	0.50		
CKD		1.03(0.86~1.23)	0.74	1.46(1.16~1.84)	0.001
Malignancy		0.63(0.50~0.79)	<0.0001		
CPOD		0.58(0.49~0.69)	<0.0001		
Asthma		0.76(0.59~0.97)	0.03	1.45(1.12~1.90)	0.006
Year of events					
2004		1			
2005		1.13(0.90~1.42)	0.29		
2006		1.44(1.16~1.79)	0.0011		
2007		1.53(1.23~1.91)	0.0002		
2008		1.78(1.44~2.21)	<0.0001		
2009		1.85(1.49~2.30)	<0.0001		
2010		1.86(1.49~2.32)	<0.0001		
2011		1.73(1.38~2.17)	<0.0001		
Epinephrine dose (per mg)		0.79(0.78~0.80)	<0.0001	0.80(0.79~0.81)	<0.0001
Vasopressin		1.04(0.12~4.07)	0.72		
Resuscitation in medical centre		1.91(1.70~2.14)	<0.0001	1.38(1.19~1.61)	<0.0001
Coronary angiography		66.71(56.90~78.25)	<0.0001	32.86(27.11~39.83)	<0.0001
Hypothermia		11.28 (6.92~18.14)	<0.0001		

Regression to age, gender, underlying diseases, Charlson comorbidity index, epinephrine dose, vasopressin use, hospital level, urbanization level, year of event, coronary angiography.

OD: odds ratio, CI: confidence interval.

a CCI: Charlson comorbidity index; DM: diabetes mellitus; CAD: coronary artery disease; HF: heart failure; Af: atrial fibrillation; CKD: chronic kidney disease; COPD: chronic obstructive pulmonary disease.

**Table 4:** Bootstrap sensitivity analysis of the logistic regression model for survival to ICU admission and hospital discharge outcomes in out-of-hospital cardiac arrest.

	Survival to ICU admission		Survival to discharge	
	Adjusted OR (95% CI)	*P* value	Adjusted OR (95% CI)	*P* value
Primary analysis				
Both	4.05(4.04~4.07)	<0.0001	4.17(4.14~4.20)	<0.0001
Amiodarone	2.23(2.23~2.24)	<0.0001	2.72(2.70~2.73)	<0.0001
Lidocaine	2.33(2.32~2.34)	<0.0001	2.51(2.48~2.53)	<0.0001
Neither	Reference		Reference	

Excluding patients with pre-existing malignancy				
Both	4.15(4.13~4.17)	<0.0001	4.14(4.11~4.17)	<0.0001
Amiodarone	2.32(2.31~2.32)	<0.0001	2.71(2.70~2.73)	<0.0001
Lidocaine	2.51(2.50~2.52)	<0.0001	2.63(2.60~2.65)	<0.0001
Neither	Reference		Reference	

Excluding patients receiving no epinephrine				
Both	3.97(3.95~3.99)	<0.0001	4.43(4.39~4.46)	<0.0001
Amiodarone	2.16(2.16~2.17)	<0.0001	2.81(2.80~2.83)	<0.0001
Lidocaine	2.19(2.18~2.20)	<0.0001	2.41(2.39~2.44)	<0.0001
Neither	Reference		Reference	

Excluding patients receiving epinephrine 0 or 1 mg				
Both	3.86(3.85~3.88)	<0.0001	4.38(4.34~4.11)	<0.0001
Amiodarone	2.12(2.11~2.12)	<0.0001	2.86(2.84~2.87)	<0.0001
Lidocaine	2.11(2.10~2.12)	<0.0001	2.46(2.43~2.49)	<0.0001
Neither	Reference		Reference	

OR: odds ratio, CI: confidence interval.

**Table 5:** Subgroup analysis of epinephrine dosage related to outcome of one-year survival^⁎^.

Epinephrine dosage	Both	Amiodarone	Lidocaine	Neither
<=5 mg	2.60(1.72~3.93)	2.36(1.88~2.96)	2.06(1.34~3.16)	Reference
P value	<0.0001	<0.0001	0.0009	
6–10 mg	2.47(1.54~3.98)	2.37(1.79~3.14)	1.83(1.01~3.32)	Reference
P value	0.0002	<0.0001	0.047	
11–15 mg	2.51(1.45~4.33)	1.18(0.78~1.80)	1.31(0.56~3.05)	Reference
P value	0.001	0.43	0.54	
>15 mg	1.46(0.85~2.51)	1.33(0.88~2.02)	1.18(0.46~2.98)	Reference
*P* value	0.17	0.17	0.73	

*P* value for interaction <0.0001.
